# Optimal telework frequency in terms of sleep and labor productivity depends on the workers’ psychological distress: A cross-sectional study in Tokyo, Japan

**DOI:** 10.1371/journal.pone.0286699

**Published:** 2023-06-14

**Authors:** Yuuki Matsumoto, Kunitaka Kumadaki, Ayako Hino, Osamu Itani, Yuichiro Otsuka, Yoshitaka Kaneita

**Affiliations:** 1 Department of Social Medicine, Division of Public Health, Nihon University School of Medicine, Tokyo, Japan; 2 Department of internal medicine, University of Occupational and Environmental Health, Japan, Kitakyushu, Japan; 3 Department of Mental Health, Institute of Industrial Ecological Sciences, University of Occupational and Environmental Health, Kitakyushu, Japan; The Hong Kong Polytechnic University, HONG KONG

## Abstract

**Objectives:**

This study aimed to determine that workers’ sleep and labor productivity differ with the telework frequency and that the optimal telework frequency depends on workers’ psychological distress.

**Methods:**

A cross-sectional study using an online-based questionnaire was conducted with 2,971 workers employed by Japanese companies from October to December 2021. We used the 6-item Kessler Scale, K6, as a nonspecific psychological distress scale to screen mental health conditions. A score of ≤ 4 was defined as low psychological distress (LPD), and a score of ≥ 5 was defined as high psychological distress (HPD). We used the Athens Insomnia Scale (AIS) as a measure of sleep quality. The Utrecht Work Engagement (UWES) and Work Functioning Impairment (WFun) scales were used as measures of labor productivity. Series of analysis of covariance (ANCOVA) was used for the data analysis.

**Results:**

The analysis included 2,013 participants (1,390 men and 623 women; mean age 43.2 years, standard deviation 11.3). Multiple comparison tests showed that among the participants categorized HPD (HPD type), the AIS estimates were lowest in the 1–2 d/wk group, with significant differences between the 0–3 d/m and ≥ 5 d/wk groups. UWES estimates were lowest in the 3–4 d/wk group with significant differences between the participants categorized LPD (LPD type) and HPD type, while no significant differences were observed among the LPD type. The WFun estimates among the LPD type decreased significantly with increasing telework frequency, while no significant differences were observed among the HPD type.

**Conclusions:**

The optimal telework frequency for sleep and labor productivity may depend on the workers’ psychological distress. The finding of this study could make a great contribution to occupational health activities and health promotion for teleworkers, which is necessary to make teleworking a sustainable work style.

## Introduction

The coronavirus disease 2019 (COVID-19) pandemic had a great impact on our work style as well as lifestyle. In Japan, the Prime Minister declared a state of emergency and requested people to telework and refrain from going out in 2020. Tokyo, in particular, the capital of Japan, has a high population density, and is known for its extremely crowded commuter trains during the morning and evening rush hours. Prior to recommendation from the World Health Organization (WHO), the Ministry of Health, Labor and Welfare (MHLW) had recommended the implementation of active teleworking to avoid the 3 Cs: crowded places, close-contact settings, confined and enclosed spaces. Although the declaration of a state of emergency has since been lifted, quite a few companies continue to recommend telework. As a result, several people who no longer need to commute, are moving out of urban centers, and the special wards have experienced an excess of out-migration for the first time in a quarter of a century. Therefore, telework has to be positioned as a sustainable rather than temporary work style, and to this end, it is necessary to pioneer occupational health activities and health promotion for teleworkers.

In addition to infection prevention, teleworking has many other advantages [1,2]. For example, the time and energy lost in commuting could instead be spent in quality time with families. However, the inconsistency of data in response to the question, “Which is better, teleworking or coming to work?” often confound us. For instance, although teleworkers report longer sleep duration, their sleep quality is worse [3,4]. In addition, in terms of productivity, teleworkers report increased concentration on work and job satisfaction; however, reports of a decline in productivity have emerged because employees who telework are unable to exercise self-control in the absence of employers monitoring their work [5–7]. A systematic review of 29 quantitative studies on telework and health issues conducted in 12 countries also reported conflicting results [8]. While telework is associated with a transition to a healthier lifestyle, it is also noted to be associated with increased sedentary time. Furthermore, they noted that mental stress may depend on individual circumstances.

Therefore, we have considered two possible primary factors that could be responsible for the differences described above. One is that few prior studies have compared the frequency of teleworking. Another is that the impact of telework varies may depend on workers’ psychological distress. Telework has immeasurable effects on mental health, such as increased stress due to the difficulty of separating work and personal life, and social isolation due to the lack of face-to-face interaction [1,9–13]. In addition, psychological distress is correlated with loneliness and less support from supervisors among Japanese teleworkers, reflecting their individual circumstances [14,15]. Therefore, we inferred that workers’ sleep and labor productivity differ with telework frequency and that these differences depend on the workers’ psychological distress.

The purpose of this study is to examine whether workers’ sleep and labor productivity differ with the telework frequency and whether optimal telework frequency depends on workers’ psychological distress. To this end, we compared health and productivity by dividing groups of workers according to their telework frequency and mental health condition. Regarding health, we focused on sleep quality (insomnia symptoms) because previous studies have reported an association with telework [4] and because it is essential to confirm employees’ sleep quality in an interview with an industrial physician when considering leave/reinstatement or work restrictions.

## Materials and methods

### Study design, participants, and ethical considerations

A cross-sectional study using an online-based questionnaire was conducted from October to December 2021. The participants were 2,971 day-shift workers employed by four companies in Tokyo who cooperated with the survey. Since the population of Tokyo is approximately 10 million, we considered a sample size of at least 1,067 persons to be sufficient, assuming a 95% confidence level, a 3% margin of error, and a population ratio of 0.5. The study’s purpose and procedures were explained to employees. Participation was voluntary, and we explained that no disadvantages would arise from not participating in the study and that the data obtained would not be used for any purpose other than the study. First of all, a survey form was created online, and the stuff in charge of each company sent the URL of the survey form to all employees via e-mail and asked them to submit their responses. Consent for participation was obtained through a web-based response and received in electronic form, and those who did not agree were not available to access and respond to any research items. Since this was an anonymous study with a substitute employee ID number, they were also explained that it was available to withdraw consent after answering the study, and that the responses would be deleted if consent was withdrawn. This study was conducted in accordance with the Helsinki declaration and its current amendments. This study was approved by the Ethics Committee of Nihon University School of Medicine (Approval No. 2021–02).

### Telework frequency and mental health condition

The question regarding frequency of teleworking was, "How much did you telework in the past month?," and the choices were categorized using a five-question method: 1) not teleworking; 2) 1 to 3 times per month; 3) 1 to 2 times per week; 4) 3 to 4 times per week; and 5) ≥ 5 times per week. Categories 1) and 2) were combined as “0 to 3 times per month” and the respondents were then divided into four groups.

We used the 6-item Kessler Scale (K6) as a nonspecific psychological distress scale to screen mental health condition [16]. K6 is a questionnaire developed to screen for depression, anxiety disorders, and other mental health conditions, and the reliability and validity of the Japanese version has been established [17,18]. It can be used in surveys of the general population and is widely used as an indicator of the degree of psychological stress; the cutoff value of K6 is 4/5 points. In this study, a score of ≤ 4 was defined as low psychological distress (LPD), and a score of ≥ 5 was defined as high psychological distress (HPD).

### Outcomes; sleep quality and labor productivity

We used Athens Insomnia Scale (AIS) as a measure of sleep quality. Utrecht Work Engagement Scale (UWES) and Work Functioning Impairment Scale (WFun) were used as measures of labor productivity.

AIS is a scale developed in accordance with the diagnostic criteria for insomnia in the International Classification of Disorders (ICD-10), and its Japanese version has been verified and validated by Okajima et al [19,20]. The questionnaire consists of eight items, with a score range of 0–24 points. The cutoff value is 5/6 points, with higher scores interpreted as stronger insomnia symptoms.

The UWES is a scale developed by Schaufeli et al [21,22]. Its Japanese version was verified and validated by Shimazu et al [23,24]. Work engagement is defined as "a positive and fulfilling psychological state related to work", assessed by three components: vitality, immersion, and enthusiasm. The three-item version was used in this study. The score range is 0–18, with higher scores interpreted as higher work motivation, i.e., higher labor productivity.

The WFun is a questionnaire developed by Fujino et al. to measure the degree of work function impairment due to health problems [25,26]. The score range is 7–35 points, with higher scores interpreted as a higher degree of work function impairment, i.e., lower labor productivity.

### Covariates

We entered basic attributes, lifestyle habits, occupational stress, location of teleworking, and duration of teleworking as covariates. Basic attributes included sex, age, presence of a roommate, occupation, employment position, commuting time, means of commuting, and work hours past a month. Lifestyle habits included exercise habits, sleep duration, alcohol consumption, caffeine intake, current smoking, use of electronic terminals outside work, and enjoyment of leisure time. Occupational stress-related factors were adapted from The Brief Job Stress Questionnaire (BJSQ) questions and included job demands, job control, supervisor support, and coworker support [27].

### Statistical analysis

The χ^2^ test was used for univariate analysis in the cross-tabulations. Series of analysis of covariance (ANCOVA) was used for the data analysis. In ANCOVA, the independent variables were telework frequency and psychological distress, and the dependent variables were AIS, UWES, and WFun. We compared the outcomes by telework frequency within the same psychological distress group (LPD or HPD) to examine whether workers’ sleep and labor productivity differ with the telework frequency. In addition to the test, we compared the outcomes between psychological distress groups (LPD vs. HPD) with the same telework frequency to examine whether optimal telework frequency depends on workers’ psychological distress. Covariate-adjusted estimates, 95% confidence intervals (95% CI), and interactions were calculated, and simple main effects tests and multiple comparison tests with Bonferroni adjustment (as needed) were performed.

Statistical analyses were performed using SPSS for Windows Version 28.0. All tests were two tailed, with *p* values < 0.05 denoting statistical significance.

## Results

Responses were obtained from 2,032 individuals. Of these, 11 who could consent to participate in the study, and 8 who had been on leave within the past month, were excluded, leaving 2,013 (1,390 men and 623 women; mean age 43.2 years, standard deviation 11.3) individuals who were included in the analysis. The valid response rate was 67.8%. Tables 1–3 show the characteristics of the participants analyzed. Regarding telework frequency, 626 participants (31.1%) teleworked 3–4 times a week most frequently. Regarding psychological distress, 1,169 (58.1%) were classified as LPH (LPD type) and 844 (41.9%) as HPD (HPD type).

**Table 1:** Characteristics of participants regarding basic attributes among four companies in Tokyo, Japan.

	Total (N = 2,013)	
Variables	n	%
**Basic attributes**		
Sex		
Male	1,390	69.1
Female	623	30.9
Age (years)		
20–29	325	16.1
30–39	437	21.7
40–49	527	26.2
50–59	609	30.3
≥ 60	115	5.7
Living alone		
No	1,454	72.2
Yes	559	27.8
Occupation		
Clerks	382	19.0
Engineers	429	21.3
Field workers	91	4.5
Creators	125	6.2
Customer service	85	4.2
Sales	782	38.8
Others	119	5.9
Employment position		
Manager or Director	129	6.4
Chief	371	18.4
Supervisor	284	14.1
Regular Employee	1,229	61.1
Commuting time		
< 90 min	1,837	91.3
≥ 90 min	176	8.7
Means of commuting		
Crowded train	1,068	53.1
Uncrowded train	513	25.5
On foot	227	11.3
Other	205	10.2
Work hours past month		
< 160 h/m	594	29.5
≥ 160 and < 200 h/m	1,062	52.8
≥ 200 h/m	357	17.7

**Table 2:** Characteristics of participants regarding lifestyle and telework among four companies in Tokyo, Japan.

	Total (N = 2,013)	
**Variables**	**n**	**%**
**Lifestyle**		
Exercise habits		
Not physically active and no intention to change	209	10.4
Not active but intending to change	499	24.8
Doing some activity but not enough	719	35.7
Regularly active but not the habit	257	12.8
Regularly active in the habit	329	16.3
Sleep duration		
≥ 7 h	1,215	60.4
< 7 h	798	39.6
Alcohol consumption		
Never or rarely	699	34.7
less than 3 d/wk	547	27.2
3–6 d/wk	393	19.5
everyday	374	18.6
Caffeine intake		
Never or rarely	175	8.7
Sometimes	358	17.8
Daily, but < twice /d	948	47.1
Daily, and > thrice /d	532	26.4
Current smoking		
No	1,472	73.1
Yes	541	26.9
Using electronic terminal outside work		
< 1 h/d	295	14.7
≥ 1 h/d and < 3 h/d	1,188	59.0
≥ 3 h/d	530	26.3
Enjoying leisure time		
Yes	1,320	65.6
No	693	34.4
**Telework**		
Frequency		
0–3 d/m	545	27.1
1–2 d/wk	436	21.7
3–4 d/wk	626	31.1
≥ 5 d/wk	406	20.2
Place		
No telework	224	11.1
Living room	843	41.9
Private room except bedroom	410	20.4
Bedroom	409	20.3
Other	127	6.3
Continuous period		
No telework	224	11.1
< 1 y	253	12.6
≥ 1 y and < 1.5 y	566	28.1
≥ 1.5 y	970	48.2

**Table 3:** Characteristics of participants regarding psychological distress among four companies in Tokyo, Japan.

	Total (N = 2,013)				
Variables	n	%	Mean	SD	Median
**Psychological distress**					
LPDs (K6 score < 5)	1,169	58.1			
HPDs (K6 score ≥ 5)	844	41.9			
**Job stressors and social supports**					
Job demands			8.6	1.9	9
Job control			8.7	1.8	9
Supervisor support			8.0	2.2	8
Coworker support			8.4	2.1	9
**K6 and outcomes**					
K6			4.6	4.5	3
AIS; Athens Insomnia Scale			4.7	3.4	4
UWES; Utrecht Work Engagement Scale			8.8	3.2	9
WFun; Work Functioning Impairment Scale			15.4	6.4	14

LPD; low psychological distress, HPD; high psychological distress.

Table 4 shows the aggregate results based on the two independent variables used in the two-way ANCOVA: telework frequency and psychological stress reaction. The highest percentage of LPD type was found when the telework frequency was ≥ 5 times per week, but the χ^2^ test showed no significant difference in the frequencies.

**Table 4:** The results of cross-tabulation and χ^2^ test based on telework frequency and psychological distress.

		Psychological distress	
		LPD type	HPD type
**Telework frequency**		n (%)	n (%)
	0–3 d/m	304 (55.8)	241 (44.2)
	1–2 d/wk	238 (54.6)	198 (45.4)
	3–4 d/wk	377 (60.2)	249 (39.8)
	≥ 5 d/wk	250 (61.6)	156 (38.4)
	*p* value	0.086	

LPD; low psychological distress, HPD; high psychological distress.

**Fig 1:**
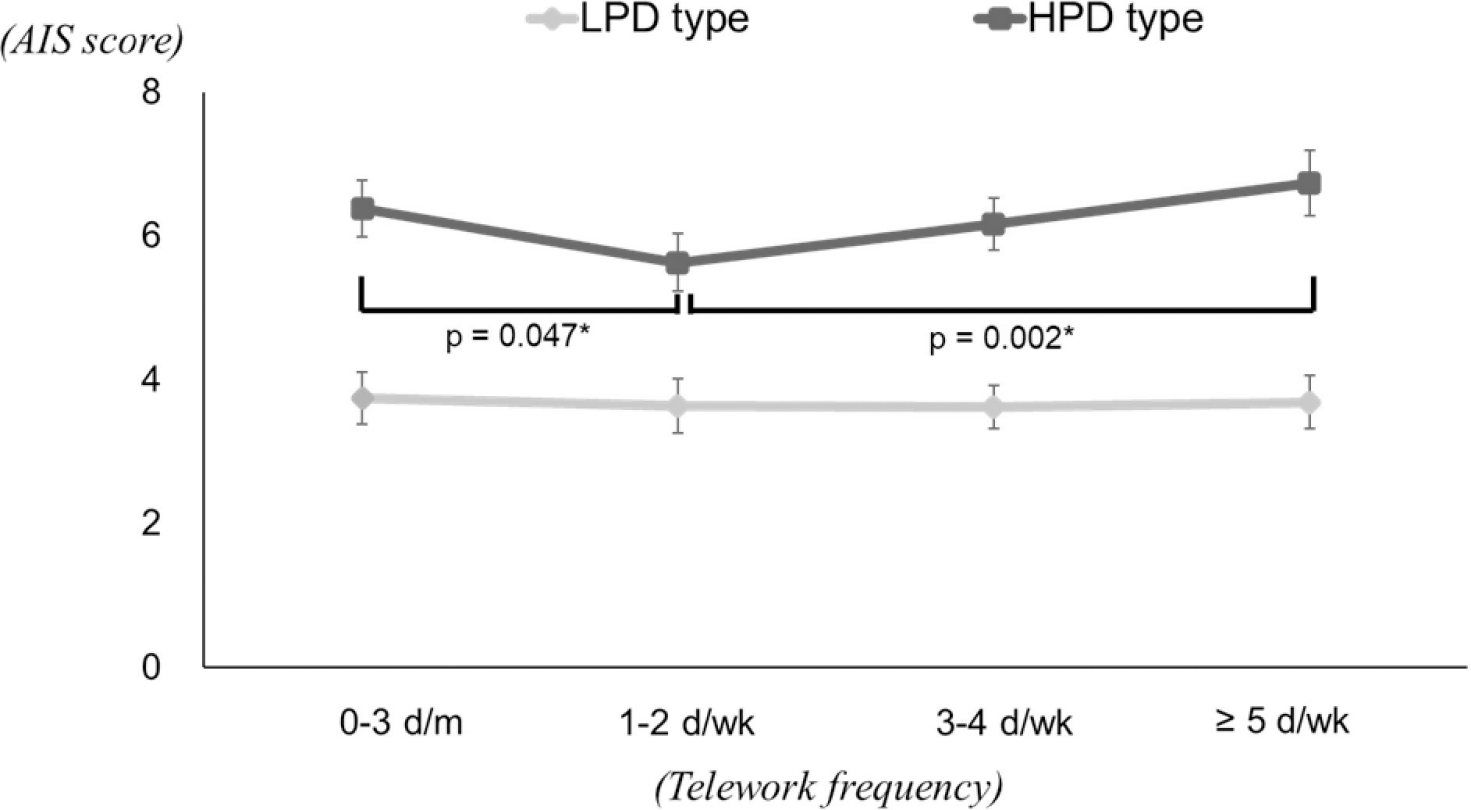
Multiple comparison tests for the AIS estimates in each psychological distress group. * Significant difference with Bonferroni adjustment.

The results of the two-way ANCOVA for AIS are shown in Table 5 and Fig 1. The value indicating an interaction was *p* = 0.065. The test for differences in psychological distress showed significant differences regardless of telework frequency, with HPD type having higher AIS estimates. In addition, tests for differences in telework frequency showed no significant differences among LPD type, while significant differences were found among HPD type. The results of multiple comparison tests among HPD type showed that the AIS estimates were lowest in the 1–2 d/wk group, with significant differences between the 0–3 d/m group and ≥ 5 d/wk group.

**Table 5:** Results of the two-way ANCOVA for AIS, UWES, and WFun scores.

		LPD type		HPD type		p value
		Estimate^†^	95% CI	Estimate^†^	95% CI	
**AIS; Athens Insomnia Scale**						
Telework frequency	0–3 d/m	3.74	3.38 to 4.09	6.38	6.00 to 6.77	< 0.001*
	1–2 d/wk	3.64	3.28 to 4.01	5.63	5.23 to 6.03	< 0.001*
	3–4 d/wk	3.62	3.33 to 3.92	6.16	5.80 to 6.52	< 0.001*
	≥ 5 d/wk	3.69	3.32 to 4.06	6.73	6.27 to 7.19	< 0.001*
*p* value		0.964		0.003**		
**UWES; Utrecht Work Engagement Scale**						
Telework frequency	0–3 d/m	8.99	8.65 to 9.34	8.77	8.39 to 9.14	0.369
	1–2 d/wk	9.18	8.83 to 9.53	8.83	8.43 to 9.22	0.188
	3–4 d/wk	8.87	8.58 to 9.15	8.11	7.76 to 8.46	< 0.001*
	≥ 5 d/wk	8.95	8.59 to 9.31	8.14	7.69 to 8.58	0.005*
*p* value		0.599		0.009**		
**WFun; Work Functioning Impairment Scale**						
Telework frequency	0–3 d/m	14.00	13.34 to 14.65	18.42	17.72 to 19.13	< 0.001*
	1–2 d/wk	13.80	13.13 to 14.47	17.89	17.15 to 18.62	< 0.001*
	3–4 d/wk	13.11	12.57 to 13.65	18.39	17.73 to 19.04	< 0.001*
	≥ 5 d/wk	12.57	11.89 to 13.25	18.62	17.77 to 19.46	< 0.001*
*p* value		0.015**		0.573		

* Significant difference between LPD type vs. HPD type.

** Significant difference among four groups divided by telework frequency.

†Adjusted sex, age, living alone, occupation, employment position, commuting time, means of commuting, work hours past 1 month, exercise habits, sleep duration (for UWES and WFun scores), drinking alcohol, taking caffeine, current smoking, using electronic terminal outside work, enjoying leisure time, telework place, telework continuous period, job demands, job control, supervisor support, coworker support.

**Fig 2:**
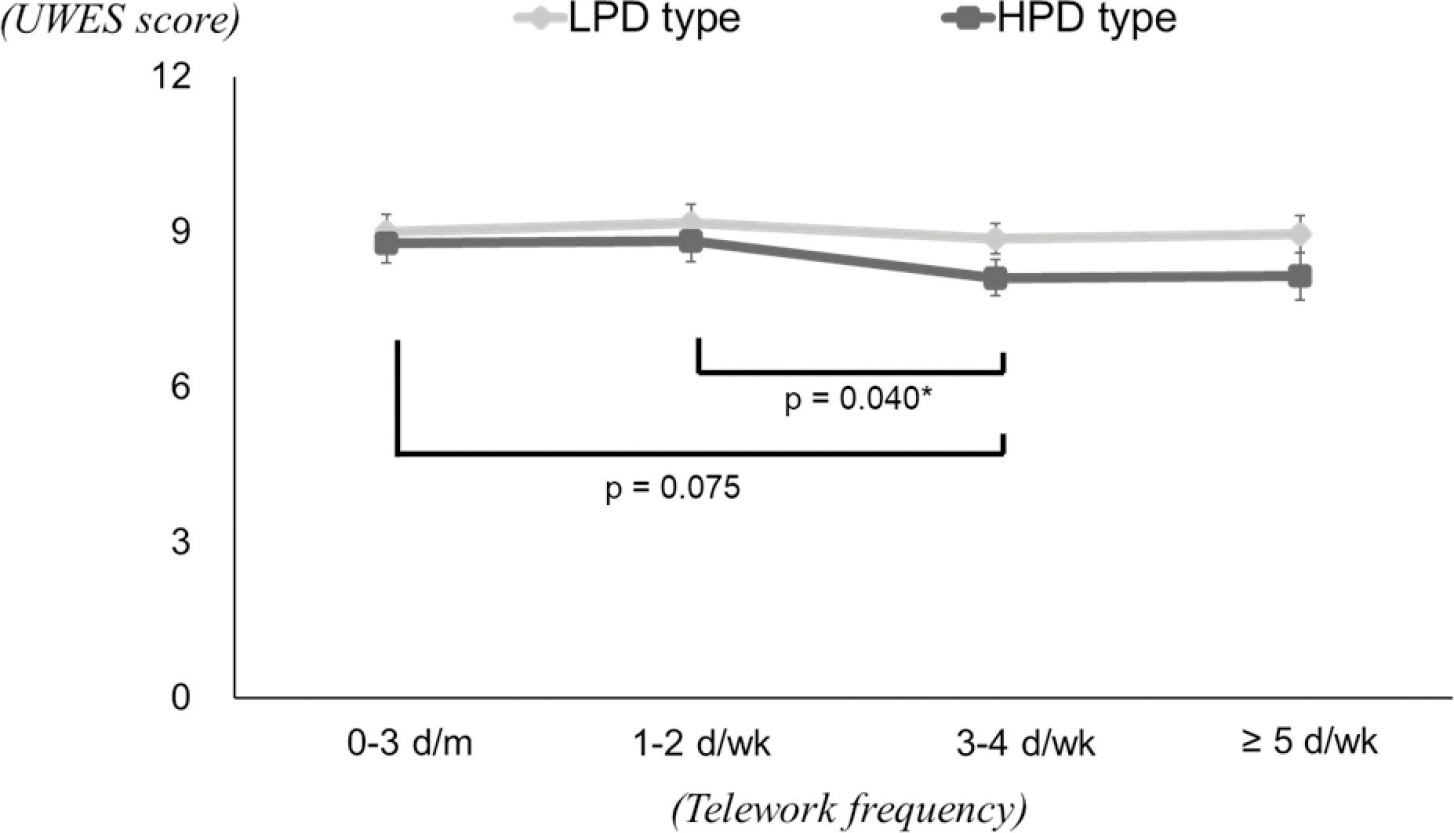
Multiple comparison tests for the UWES estimates in each psychological distress group. * Significant difference with Bonferroni adjustment.

The results of the two-way ANCOVA for UWES are shown in Table 5 and Fig 2. The value indicating an interaction was *p* = 0.246. In the test for differences in psychological distress, significant differences were found in the 3–4 d/wk and *≥* 5 d/wk groups, with the UWES estimate being lower in the HPD type than in the LPD type. The test for differences in telework frequency showed no significant differences among LPD type, but significant differences among HPD type. As in the results of multiple comparison tests among LPD type, the UWES estimates were lowest in the 3–4 d/wk group, with significant differences between the 1–2 d/wk group.

**Fig 3:**
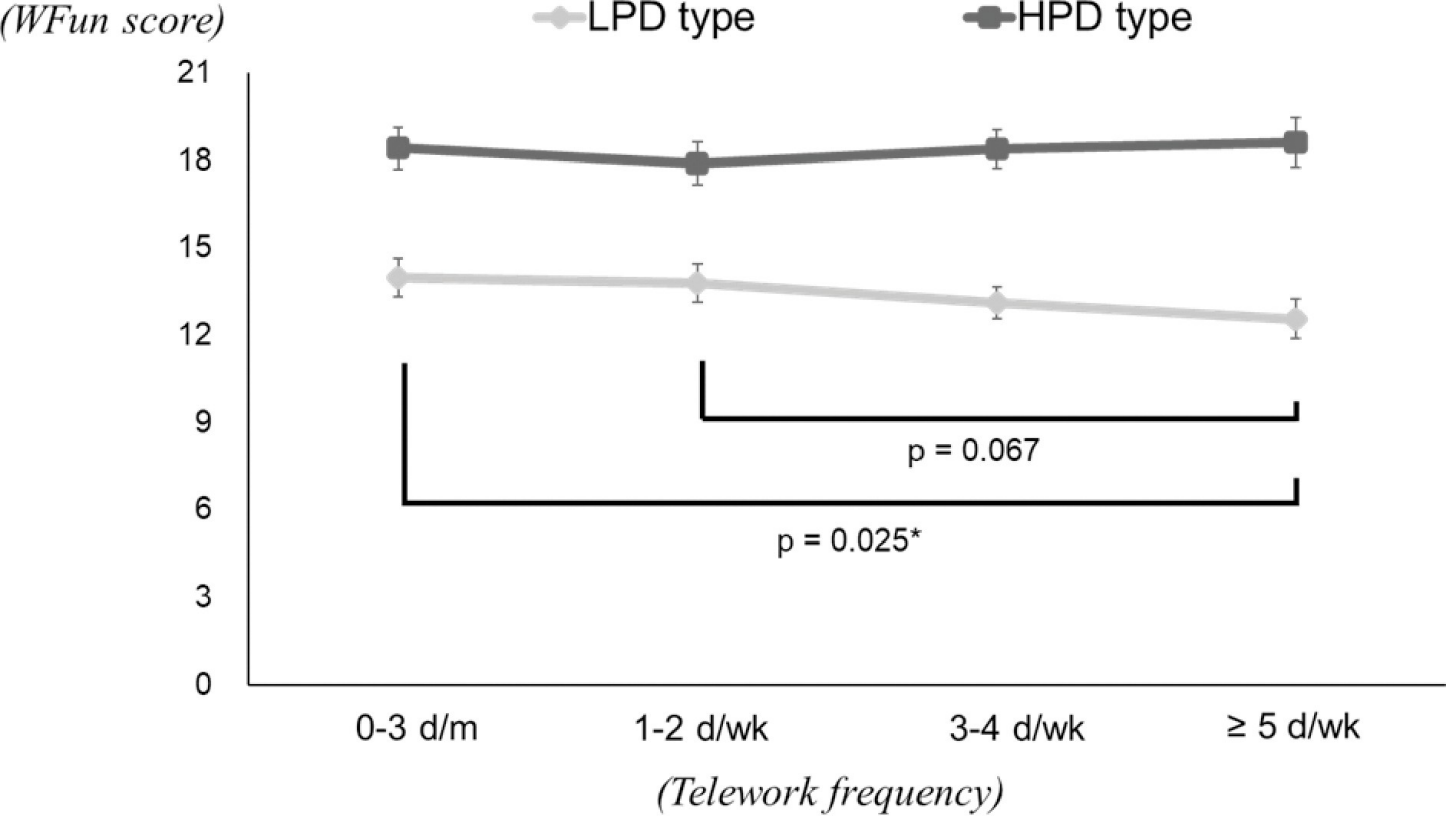
Multiple comparison tests for the WFun estimates in each psychological distress group. * Significant difference with Bonferroni adjustment.

The results of the two-way ANCOVA for WFun are shown in Table 5 and Fig 3. The value indicating an interaction was *p* = 0.026. The test for differences in psychological distress showed significant differences regardless of telework frequency, with all HPD type having higher WFun estimates. The test for differences in telework frequency showed no significant differences among HPD type, but significant differences among LPD type, with WFun estimates decreasing with increasing telework frequency. The results of multiple comparison tests among LPD type showed that the WFun estimates were lowest in the ≥ 5 d/wk group, which differed significantly from the 0–3 d/m group.

## Discussion

The purpose of this study is to demonstrate that workers’ sleep and labor productivity differ with the telework frequency and that the optimal telework frequency depends on workers’ psychological distress. As the results of this study, clear differences were observed in workers’ sleep quality and labor productivity by the telework frequency, which differed according to the level of workers’ psychological distress. It was suggested that there is an optimal telework frequency at which sleep quality and labor productivity are the most favorable, and that it may depend on workers’ level of psychological distress. These findings are novel, and a strength of the present study is that the sample population could be classified based on telework frequency and psychological distress without a bias in the number of participants. The findings are very important for creating a sustainable hybrid work style that combines coming to work and teleworking.

Although 58.1% of the participants in this study had K6 scores < 5, the percentage of those with K6 scores < 5 was over 65% for those aged 20–59 years in the 2019 Comprehensive Survey of Living Conditions [28]. Thus, the results in this study report a lower rate than the national average. However, since 2019 was before the COVID-19 pandemic, we considered it difficult to simply compare them.

For all outcomes, there were significant differences in either LPD type or HPD type by telework frequency. This suggests that workers’ sleep and labor productivity differ by telework frequency. As teleworkers spend more time sitting and less time being physically active [29], there is an association between physical inactivity and increased risk of sleep disorders [30]. In addition, exposure to bright light during the day improves sleep quality, suggesting that coming to work may alleviate insomnia symptoms [31]. Nevertheless, as mentioned earlier, teleworking has the advantage of making it easier to secure sleep time. In LPD type, AIS score was better among the 1–2 d/wk than the 0–3 d/m group, which may be due to the combined AIS score for sleep quality and quantity. The p-value for the interaction of UWES, whereas, is above 0.1, indicating that the difference in the effect of telework frequency by the level of psychological distress is smaller than that of WFun and AIS. In WFun, the HPD type showed higher scores than LPD type, which is consistent with previous studies reporting higher WFun scores in depressed patients [32]. Among LPD type, the results were significantly different depending on the telework frequency. Because WFun can also be used as an indicator of labor productivity, more frequent teleworking in the LPD type could be interpreted as higher labor productivity.

Based on the three outcomes used in this study, the telework frequency with lower AIS and WFun scores and higher UWES scores is considered the optimal telework frequency for workers. Then, the optimal telework frequency would be ≥ 5 d/wk for the LPD group and 1–2 d/wk for the HPD group. In other words, the results suggest that the optimal telework frequency depends on the psychological distress of the workers. The physical activity and bright light during the day, as described above, effects may have been more readily apparent for the AIS score among HPD type, who had stronger insomnia symptoms than LPD type. Furthermore, the significant difference for UWES between the HPD type and LPD type when teleworking is ≥ 3 d/wk should not be overlooked. Negative feelings caused by teleworking are noted to be due to increased isolation and loneliness [11]. The isolation and loneliness may also be associated with reductions in positive emotions such as work engagement, although this effect may be more likely among HPD type than LPD type. LPD type, whereas, had the best WFun score when teleworking is ≥ 5 d/wk. Some of the WFun questions included items related to sociability and the number of interruptions from work. One participant said, "I am interrupted less often while teleworking because I don’t have to answer office telephone calls." For those with good mental health who do not suffer from teleworking problems such as poor communication, teleworking may increase work productivity by reducing the number of interruptions.

These findings suggest the need to consider workers’ psychological distress status when adjusting telework frequency. Between 2020 and 2021, 10.1% of Japanese companies had workers on leave due to mental health problems, while the percentage of workers with mental health problems at companies with ≥50 workers exceeded 25%, and companies with ≥ 500 workers exceeded 80% [33]. Work-limiting measures should be considered for workers with mental health problems. In such cases, occupational physicians and human resources staff must decide on the frequency of teleworking. Most had no experience in adjusting telework frequency for workers with mental health problems before the COVID-19 pandemic, and are currently doing so with limited experience and resources. Therefore, the findings in this study may be very useful in determining the telework frequency for workers with mental health problems.

This study has several limitations. First, there is a possible selection bias since only workers of companies in Tokyo, the capital of Japan, were included in the study. Because of Tokyo’s high population density and advanced communications technology, the benefits of telework may be more easily exposed than for workers in rural companies. Second, because the survey is entirely self-reported, there is a possible information bias that makes it less objective. Third, there could be confounding bias due to factors not entered as covariates, such as household income and medication history. In particular, those with restricted employment may be more likely to the HPD type. Finally, because it is a cross-sectional survey, temporal associations could not be demonstrated.

## Conclusions

A novel finding in this study was that the optimal telework frequency for sleep and labor productivity may depend on the workers’ psychological distress. Specifically, sleep quality and work engagement differed by telework frequency among high psychological distress workers, with 1–2 days/week being the most favorable, whereas these did not differ by telework frequency among low psychological distress workers. On the other hand, work dysfunction differed by telework frequency only among low psychological distress workers, with more frequent telework resulting in lower work dysfunction. Further study is required to demonstrate the temporal relevance of the effects of telework. However, the finding of this study could make a great contribution to occupational health activities and health promotion for teleworkers, which is necessary to make teleworking a sustainable work style.
